# Antiviral Effect of Bovine Lactoferrin against Enterovirus E

**DOI:** 10.3390/molecules27175569

**Published:** 2022-08-29

**Authors:** Małgorzata Wróbel, Joanna Małaczewska, Edyta Kaczorek-Łukowska

**Affiliations:** Department of Microbiology and Clinical Immunology, Faculty of Veterinary Medicine, University of Warmia and Mazury in Olsztyn, Oczapowskiego Street 13, 10-719 Olsztyn, Poland

**Keywords:** bovine lactoferrin, enterovirus E, antiviral

## Abstract

Enterovirus E (EV-E), a representative of the Picornaviridae family, endemically affects cattle across the world, typically causing subclinical infections. However, under favorable conditions, severe or fatal disorders of the respiratory, digestive, and reproductive systems may develop. There is no specific treatment for enterovirus infections in humans or animals, and only symptomatic treatment is available. The aim of this study was to determine the in vitro antiviral effect of bovine lactoferrin (bLF) against enterovirus E using virucidal, cytopathic effect inhibition, and viral yield reduction assays in MDBK cells. The influence of lactoferrin on the intracellular viral RNA level was also determined. Surprisingly, lactoferrin did not have a protective effect on cells, although it inhibited the replication of the virus during the adsorption and post-adsorption stages (viral titres reduced by 1–1.1 log). Additionally, a decrease in the viral RNA level in cells (by up to 75%) was observed. More detailed studies are needed to determine the mechanism of bovine lactoferrin effect on enterovirus E. However, this highly biocompatible protein ensures some degree of protection against infection by bovine enterovirus, which is particularly important for young animals that receive this protein in their mother’s milk.

## 1. Introduction

Enterovirus E (EV-E) belongs to the Picornaviridae family, which is comprised of small, nonenveloped, icosahedral viruses that have a positive-sense, single-stranded RNA. This virus was isolated from cattle faeces in the 1950s and, similar to many human and animal enteroviruses, was initially classified as an enteric cytopathogenic orphan virus (ECBO, enteric cytopathogenic bovine orphan). The reason for this was that these viruses were isolated from healthy specimens and could not be unambiguously linked to any known pathological syndrome [1]. EV-E appears endemically in populations of cattle across the world, and the shedding of large amounts of the virus occurs in animal faeces. This virus spreads through the fecal–oral route and typically causes subclinical infections. However, under advantageous conditions, it can result in disorders of the respiratory, digestive, and reproductive systems with a severe and sometimes fatal course [2,3,4,5]. It is also classified as a member of a large group of pathogens involved in the pathogenesis of the bovine respiratory disease complex (BRDC), a complex, multifactorial disease responsible for large economic losses in cattle breeding [6]. In addition to having a global distribution, enterovirus E invades a broad spectrum of hosts, such as cattle, small ruminants, and animals living in the wild, while the presence of antibodies against EV-E has been confirmed in horses, dogs, donkeys, and humans [7,8,9,10,11]. Because enteroviruses are characterized by a high frequency of mutations and recombination, they are highly capable of evolving over time, which is linked to the risk of the zoonotic potential of animal enteroviruses [12]. In the human population, the percentage of EV-E seropositive patients in rural areas is approximately the same as that in urban areas, which attests to the fact that the virus is relatively widespread in the environment [11]. It can also survive in the environment longer than other known bovine viruses, due to the high stability of enteroviruses and their tolerance to changes in pH, high temperature, salinity and many chemical agents [10]. It is also thought that atmospheric precipitations can promote the spread of enteroviruses in the environment, and large amounts of these viruses in water are treated as an indicator of water contamination with faeces [7,13]. For all the above reasons and pursuant to the EU standard [14], enterovirus E is used in studies on the activity of chemical disinfectants and antiseptics used in the veterinary area.

Due to the specificity of viral infections, the use of antiviral compounds in medicine is very limited compared to antibacterial or antifungal chemotherapy. According to the Centers for Disease Control and Prevention (CDC), there is no specific treatment for nonpolio enterovirus infection in humans, and only symptomatic treatment is administered [15]. The use of antiviral chemotherapy in veterinary medicine is far less available than in human medicine, especially in the case of food species. Only a few antivirals used in human medicine have been adapted to veterinary clinical medicine, such as acyclovir in feline ocular infections caused by feline herpesvirus 1 [16]. As in the case of human enteroviruses, there is no treatment for enterovirus infections in animals.

Lactoferrin is a multifunctional glycoprotein from the transferrin family that is present in body fluids, excretions (including colostrum and milk), and neutrophil granules. It is a significant humoral factor of innate immunity, and its role in infections and inflammatory states of the organism arises from both its antimicrobial and immunomodulatory activities [17]. Lactoferrin shows antiviral activity against both DNA and RNA viruses, acting particularly during the early stages of an infection by binding to the particles of some viruses or blocking the cellular receptors for the viruses. Lactoferrin has also been observed to inhibit the replication of some viruses in infected cells and to enhance the antiviral immunological response of the organism [18,19]. The viruses sensitive to lactoferrin activity include enteroviruses, such as enterovirus 71, coxsackieviruses, and the human viruses ECHO 5 and 6 [19]. It is also suspected that breast-feeding ensures protection from enterovirus infection in children due to the presence of both maternal antibodies in milk and factors associated with nonspecific humoral resistance, such as lactoferrin [20,21]. This observation seems to be confirmed by the experiment conducted by Chen et al. [22] in mice, in which recombinant lactoferrin present in murine milk protected pups from lethal enterovirus 71 infection.

The purpose of this study was to determine the antiviral activity of bovine lactoferrin against enterovirus E under in vitro conditions. To date, the antiviral activity of this biocompatible protein against bovine enterovirus has not been tested.

## 2. Results

### 2.1. Choice of Lactoferrin Concentrations Tested in the Experiment

When selecting the concentrations of lactoferrin tested in this study, we were guided by the results of our earlier studies, which were conducted using the same cell line (MDBK) and the same batch of bovine lactoferrin purchased from the same manufacturer (Sigma-Aldrich, Schnelldorf, Germany). The 50% cytotoxic concentration (CC_50_) of lactoferrin was 4.907 mg/mL, and the maximum tolerable concentration (MTC) was 2.5 mg/mL [23]. As in previous studies and according to the literature data, the concentrations of 1, 0.5, 0.25, 0.125, and 0.06 mg/mL bovine lactoferrin were chosen for further testing.

### 2.2. Cytopathic Effect Inhibition and Virucidal Activity of Bovine Lactoferrin against Enterovirus E

**Figure 1:**
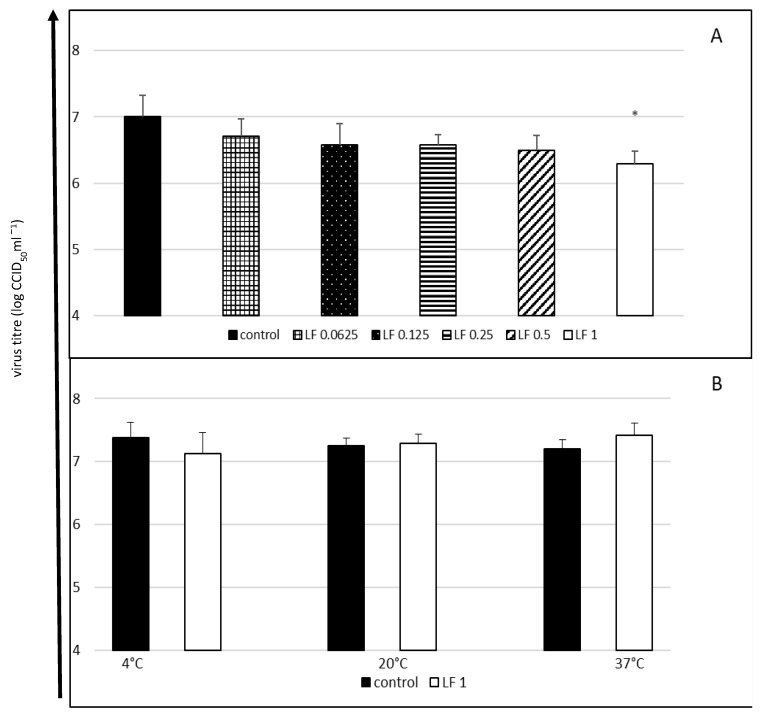
Cytophatic effect inhibition (**A**) and virucidal activity (**B**) of bovine lactoferrin against enterovirus E. The 50% endpoint virus titres (CCID_50_) were determined using the Reed and Muench method. Lactoferrin (LF) concentrations in mg/mL; control: untreated virus. Data presented as means ±SD (standard deviation) for three independent experiments (*n* = 3). Statistically significant differences between control and treatments marked with an asterisk at * *p* < 0.05.

In the first experiment, in which both lactoferrin and the virus were incubated with cells throughout the entire experiment, only the highest concentration of lactoferrin considerably reduced the final titre of the virus (a decrease in the titre by 0.71 log, i.e., by approximately 63%) (Figure 1A).

The highest tested lactoferrin concentration did not have a direct virucidal effect on EV-E after 60 min of contact, regardless of the incubation temperature (Figure 1B).

### 2.3. Viral Yield Reduction in the Presence of Bovine Lactoferrin—A Time-of-Addition Assay

**Figure 2:**
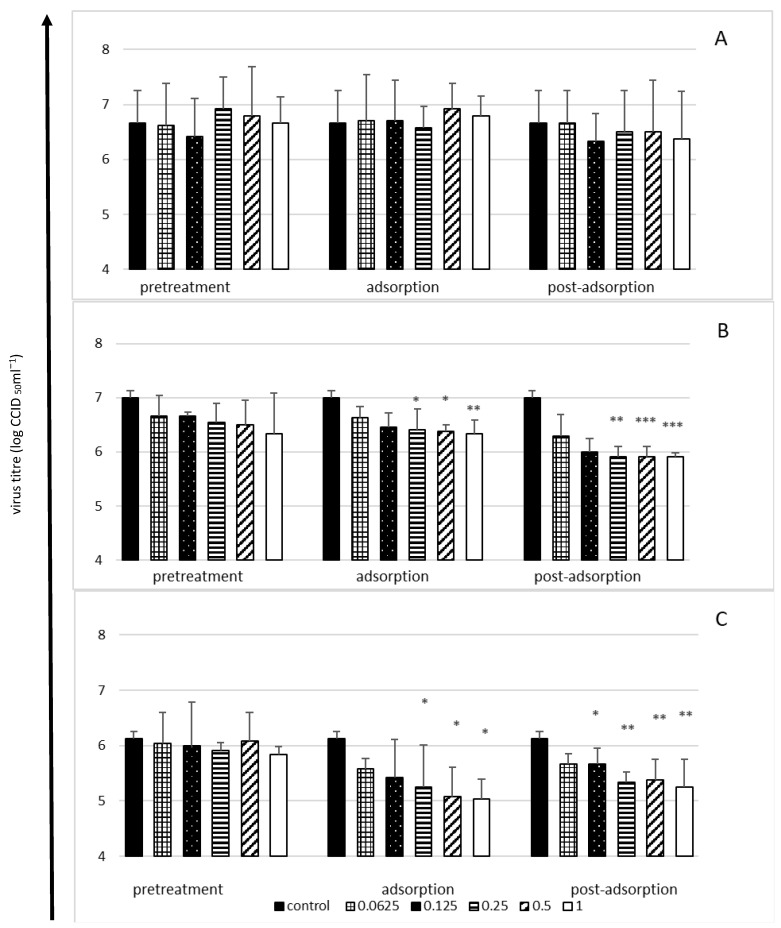
Viral yield reduction by lactoferrin—time-of-addition assay. Cells infected with high (MOI = 10) (**A**), medium (MOI = 1) (**B**) or low (MOI = 0.1) (**C**) infectious doses of enterovirus E. Cells pretreated with lactoferrin for 2 h before infection (pretreatment); lactoferrin present during virus adsorption (adsorption); lactoferrin added just after virus adsorption (post-adsorption). Culture supernatants collected for virus titration (CCID_50_) after 24 h of incubation. Lactoferrin (LF) concentrations in mg/mL; control: untreated cells. All data expressed as means ±SD (standard deviation) for three independent experiments (*n* = 3). Asterisks refer to statistically significant differences between control and lactoferrin-treated cells at: * *p* < 0.05, ** *p* < 0.01, *** *p* < 0.00.

The antiviral activity of lactoferrin was only observed in the case of the low and medium infection doses of the virus (multiplicity of infection, MOI = 0.1 or 1, respectively), whereas when the infection dose was high (MOI = 10), lactoferrin did not have a significant effect on the production of virus progeny by cells (Figure 2).

Irrespective of the dose of the virus, bLF did not have a protective effect on cells (pretreatment stage), although an inhibitory effect was observed both at the adsorption stage and immediately after the adsorption of the virus (Figure 2). The highest reduction of the viral titre (by 1–1.1 log, i.e., ca. 90%) was observed in the case of the lowest infection dose of the virus at the adsorption stage (Figure 2C) and at the post-adsorption stage when the medium dose was tested (Figure 2B), with a broader range of bLF concentrations showing antiviral activity at the post-adsorption stage (Figure 2B,C).

### 2.4. Effect of Lactoferrin on Viral RNA Load in Enterovirus E-Infected Cells

**Figure 3:**
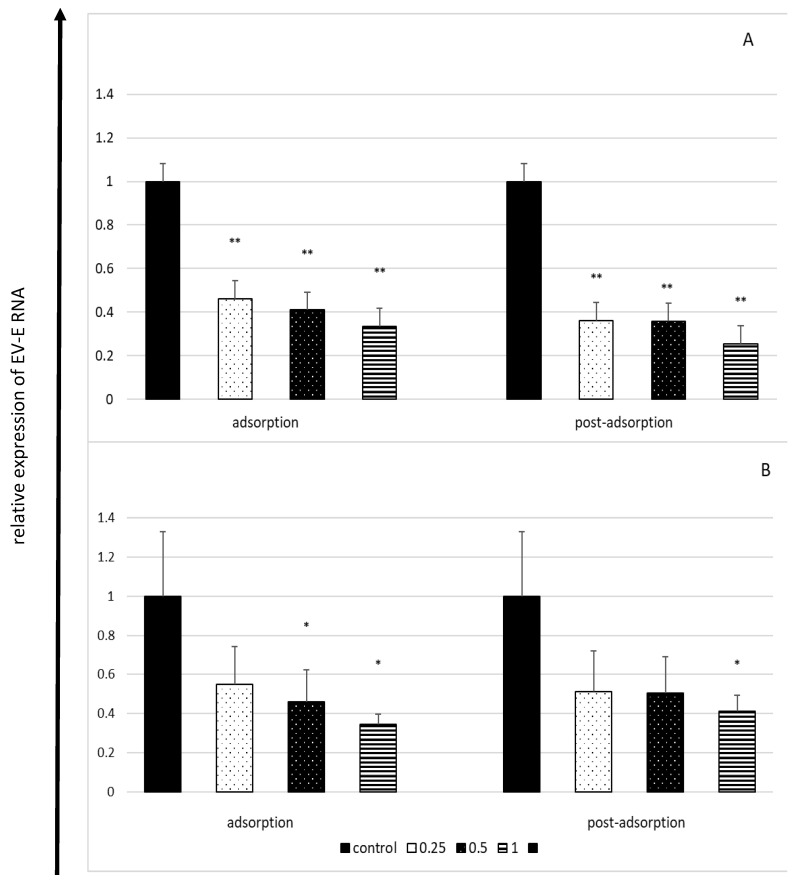
Effect of lactoferrin on viral RNA load in enterovirus E-infected cells after 6 h (**A**) and 18 h (**B**) of incubation (RT-qPCR). Lactoferrin (LF) concentrations in mg/mL; control: untreated cells. Lactoferrin present only during virus adsorption (adsorption) or added just after virus adsorption (post-adsorption). Amounts of intracellular enterovirus E RNA from control (lactoferrin-untreated) cells set as 1, results obtained from treated cells expressed as the relative amount of the control virus RNA. All data expressed as means ±SD (standard deviation) for three independent experiments (*n* = 3). Asterisks refer to statistically significant differences between control and lactoferrin-treated cells at: * *p* < 0.05, ** *p* < 0.01.

The effect of lactoferrin on the quantity of the viral intracellular RNA was weaker than its effect on extracellular virus titres. This effect was dose-dependent and inversely proportional to the incubation time. The most distinct reduction observed in the amount of viral RNA (by approximately 75%) was observed after 6 h of incubation (Figure 3).

## 3. Discussion

In the absence of registered enterovirus-specific treatments, the search for antivirals against human enteroviruses has gained importance [24,25,26]. Bovine enterovirus E has not been a frequent subject of studies on the activity of potential antiviral compounds. In recent years, only a team of researchers from Poland has been testing the virucidal activity of various betulin derivatives against this virus [27,28]. However, there have been recent cases of severe infections in cattle, which have been identified as being caused by bovine enterovirus [2,4,5,29,30]. This is probably a consequence of improved testing techniques in veterinary medicine, which may force us to revise our view of EV-E as a harmless, almost commensal cattle virus. In view of the above facts, we decided to analyze the antiviral potential of substances of natural origin, namely, lysozyme, lactoferrin and nisin, against EV-E in our laboratory. The preliminary study showed that only lactoferrin demonstrated a satisfactory level of activity against EV-E and was therefore chosen for further tests. The other substances did not demonstrate a considerable influence on enterovirus E (data unpublished).

In in vitro experiments on the antiviral activity of bovine lactoferrin against human enteroviruses, this protein has been confirmed to act against enterovirus 71, coxsackieviruses, echovirus 5 and 6 and poliovirus type 1 [21,31,32,33,34,35], with quite a wide range of active concentrations. The lowest IC_50_ values (50% inhibitory concentration) of lactoferrin were noted against coxsackievirus (9.3 µg/mL) [31] and enterovirus 71 (10–34.5 µg/mL) [31,32], whereas the IC_50_ was 1.56 µM, i.e., ca. 50 µg/mL, against echovirus 6 [21] and as high as 650 µg/mL against poliovirus type 1 [35]. In some studies, the authors used predetermined concentrations of LF in a range of 12.5 µM (i.e., ca. 0.39 mg/mL) to 2 mg/mL [33,34,35]. A concentration of 250 µg/mL produced a 73% inhibitory effect against enterovirus 71 [32], while 2 mg/mL inhibited poliovirus type 1 by 75–90%, depending on the stage of viral replication [35]. The 63% reduction in the final titre of EV-E by bLF at a concentration of 1 mg/mL and 90% reduction in the virus titres in the time-of-addition assay at concentrations within 0.125–1 mg/mL bLF that were documented in our study fall within the broad ranges of lactoferrin activity described by other authors. The effect of lactoferrin on the viral RNA load in enterovirus E-infected cells corresponded with the results of the time-of-addition assay, although it was not as strongly observed. It is not easy to explain this finding, as the available literature lacks a similar comparison regarding human enteroviruses. None of the cited studies included the isolation of viral RNA or real-time PCR analysis.

As mentioned previously, the antiviral activity of lactoferrin against most viruses takes place at the early stages of infection and is a consequence of lactoferrin’s interaction with virus particles or with the cell, while the inhibition of the intracellular stages of viral replication by lactoferrin has been described far less often [18,19]. Enteroviruses are not an exception in this regard. However, the research results provided by different authors concerning the ability of bovine lactoferrin to bind to viral proteins are not conclusive. LF has been demonstrated to be able to bind to the proteins of echovirus 71 [32] but is unable to bind to either intact viral particles (N form) or intermediate particles (A form) of enterovirus 6. Lactoferrin gains this ability only in an acidic (pH 4–5) environment, which is characteristic of late endosomes [33]. Thus, the cited authors observed the lack of the ability of LF to neutralize echovirus 6 at a neutral pH, which is consistent with the results of our study on enterovirus E.

In nearly all the studies cited in this paper in which human enteroviruses were tested, lactoferrin showed a protective influence on cells. Such an effect was observed in the cases of enterovirus 71 [31,32], echovirus 5 [34] and echovirus 6 [21,33]. For three decades, it has been known that interactions of lactoferrin with cells are a consequence of its ability to bind glycosaminoglycans and low-density lipoprotein receptors on cell surfaces, which are utilized by various viruses as cell receptors [36]. Although cell surface receptors to EV-E have not been identified to date [37], the protective effect of lactoferrin observed in the case of human enteroviruses has substantiated the hypothesis that this protein can also protect cells from being infected with enterovirus E. With enterovirus 71, lactoferrin demonstrated a stronger protective effect the longer it was incubated with cells, and the shortest time needed to achieve this effect was 30 min [31,32]. Based on these results, we decided in our experiment to incubate MDBK cells with lactoferrin for two hours. Surprisingly, the relatively long cell pretreatment with different concentrations of LF did not produce any protective effect against EV-E, regardless of the multiplicity of infection of the virus used. In our earlier study on another bovine RNA virus (bovine viral diarrhea virus) using the same cell line, bovine lactoferrin demonstrated a protective influence on cells, but the observed effect, typically reaching the highest level after the shortest time period tested, which was 24 h, was weakened when the incubation time was prolonged [23]. In the present study, because the enteroviral life cycle is rapid and virus progeny are usually released after 8 h [38], titres of EV-E in the time-of-addition assay were determined only after 24 h. Nonetheless, the incubation time in our study might have been too long to observe the protective action of LF towards the cells.

The antiviral activity of bovine lactoferrin at the stage of the adsorption of the virus observed against bovine enterovirus in our experiment is totally consistent with the results of studies reported by other authors who tested the activity of this protein against human enteroviruses [21,33,34,35]. What surprised us, however, was the powerful antiviral effect of bLF against EV-E at the post-adsorption stage, while no inhibitory effect of LF at this stage of infection against poliovirus type 1, echovirus 5, and enterovirus 71 has been documented [31,34,35]. In turn, Weng et al. [32] showed that it is necessary for this protein to enter cells no later than 60 min after the adsorption of the virus has been completed to achieve the inhibitory effect of lactoferrin against enterovirus 71. The strong inhibitory effect of LF against echovirus 6 after adsorption has been noted by Pietrantoni et al. [21]. These researchers performed a more detailed analysis of this effect in their subsequent experiment, thereby demonstrating that both LF and echovirus 6 enter cells by endocytosis and that the virus-lactoferrin interaction takes place in an acidic environment of endosomes inside the cell [33]. Thus, the cited scholars described novel mechanisms of the antiviral activity of lactoferrin, consisting of the prevention of virus uncoating and prevention of the viral eclipse phase. In our experiment, lactoferrin was added to the cells immediately after viral adsorption was completed and remained present in the medium until the termination of the experiment (24 h). Hence, it could have interfered with virus uncoating. However, it is not known whether enterovirus E can penetrate cells via endocytosis or if it takes advantage of another entry pathway. Analysis of the mechanism of the antiviral activity of lactoferrin against EV-E requires further investigation.

## 4. Materials and Methods

### 4.1. Cells, Virus and Bovine Lactoferrin

Madin-Darby bovine kidney (MDBK, ATCC CCL-22) cells were cultured at 37 °C in a humidified atmosphere with 5% CO_2_ in Dulbecco’s modified Eagle’s medium (DMEM) supplemented with 10% horse serum, 1% nonessential amino acid solution and 1% antibiotic-antimycotic solution (all medium components were purchased from Sigma-Aldrich, Schnelldorf, Germany).

Enterovirus E (LCR4 strain, ATCC VR-248) was propagated in MDBK cells. To prepare a stock virus for titration, a confluent monolayer of MDBK cells grown in a 75-cm^2^ flask was inoculated with a virus stock at a 1:10 virus dilution in maintenance medium (containing 2% horse serum instead of 10%) and incubated. When an extensive cytopathic effect (CPE) was observed, the infected cells were centrifuged, and the aliquoted supernatant was stored at −80 °C until use. This virus stock was titred by an end-point dilution assay (10-fold serial dilutions of virus) on MDBK cells grown in 96-well plates (eight wells per dilution). Three days after inoculation, the cytopathic effect was recorded using an inverted phase contrast microscope. The 50% endpoint virus titres (CCID_50_, 50% cell culture infective dose) were calculated using the Reed and Muench method [39].

Lactoferrin from bovine milk, purchased from Sigma-Aldrich, was tested for anti-enterovirus E activity. Just before use, the lactoferrin was dissolved in DMEM at final protein concentrations of 0 (control cells), 0.0625, 0.125, 0.25, 0.5, and 1 mg/mL. When selecting the concentrations of lactoferrin tested in this study, we were guided by the results of our earlier studies, which were conducted using the same cell line (MDBK) and the same batch of bovine lactoferrin [23].

### 4.2. Antiviral Assays

Since there are no registered specific anti-enterovirus drugs, there was none to use as a positive control for comparisons in the antiviral assays.

#### 4.2.1. Cytopathic Effect Inhibition Assay

To confirm the potential anti-enterovirus E activity of bovine lactoferrin, its effect on the final virus titre was evaluated. The virus was titrated in the presence of all tested concentrations of lactoferrin, as described above. The control virus was titrated in compound-free medium. In this experiment, both virus and lactoferrin were present in the medium during the entire incubation period (72 h). All experiments were repeated three times.

#### 4.2.2. Virucidal (Virus Neutralisation) Assay

To evaluate the potential virucidal activity of bovine lactoferrin, the stock virus was incubated with the highest tested concentration of lactoferrin (1 mg/mL) in DMEM (the stock virus final dilution 1:10) under different experimental conditions (contact time, 60 min; contact temperatures, 4, 20 or 37 °C). The control virus was diluted with lactoferrin-free medium and incubated under the same conditions. Each mixture was then titrated in MDBK cells, and CCID_50_ titres of lactoferrin-treated virus were compared with control virus titres (tested under the same set of conditions). Each experiment was repeated three times.

#### 4.2.3. Yield Reduction Assay

MDBK cells were seeded in 24-well plates and grown for 24 h before infection. Then, the growth medium was replaced with maintenance medium containing enterovirus E and incubated at 37 °C for 1 h (adsorption). Afterwards, the inoculum was removed, the cells were washed three times with medium, and fresh maintenance medium was added to the wells. In this experiment, three different infectious doses (multiplicity of infection, MOI) of enterovirus E were tested: high (MOI = 10), medium (MOI = 1) and low (MOI = 0.1).

To determine the mode of antiviral activity of lactoferrin, the following experimental procedures were carried out:

- Cell pretreatment stage: Cells were pretreated with various lactoferrin concentrations (2 h at 37 °C), washed three times with medium and then infected with virus (1 h at 37 °C)
- Adsorption stage: Various lactoferrin concentrations were added simultaneously with the virus inoculum (1 h at 37 °C), followed by washing.
- Post-adsorption stage: Various lactoferrin concentrations were added to cells just after virus adsorption (1 h at 37 °C) and were present in the medium to the end of the experiment.

After 24 h of incubation, culture supernatants were collected, and the extracellular virus was titrated (CCID_50_) in MDBK cells, as described above. All experiments were repeated three times.

### 4.3. RNA Isolation and RT-qPCR

In this experiment, only a low infectious dose (MOI = 0.1) of eneterovirus E was used, and only those experimental designs that resulted in decreased extracellular virus titres in the yield reduction assay were further tested for their effects on intracellular viral RNA synthesis. Since the enteroviral life cycle is fast and virus progeny are usually released after 8 h, we evaluated viral RNA levels after 6 and 18 h of incubation. For this purpose, the supernatants were removed, cells were washed twice with PBS, and 0.8 mL of Fenozol reagent (A & A Biotechnology, Poland) was added to each well and mixed by pipetting until complete cell lysis occurred.

Isolation of the RNA from the cell culture was carried out with the use of a Total RNA Mini kit (A&A Biotechnology, Gdynia, Poland) according to the manufacturer’s recommendations. The concentrations of eluted RNA were measured with a BioSpectrometer (Eppendorf, Hamburg, Germany). Reverse transcription was performed with the use of a High-Capacity cDNA Reverse Transcription Kit (Life Technologies, Waltham, MA, USA) following the manufacturer’s protocol. Real-time PCR was performed to identify and quantify enterovirus E. The 20-μL reaction sample contained 10 μL of DyNAmo HS SYBR Green qPCR Master Mix 2× (Life Technologies, Waltham, MA, USA), 0.5 μL of each primer at 10 μM (EV-E183 for and EV-E183 rev; Table 1), 2 μL of cDNA, and 7 μL of ribonuclease-free water. TaqMan qPCR was conducted under the following conditions: activation of the polymerase at 95 °C for 15 min followed by 40 two-stage cycles of denaturation at 94 °C for 30 s, primer annealing at 62 °C for 20 s, and chain elongation at 72 °C for 20 s.

To determine the viral copy number in the analyzed samples, a standard curve was plotted. Standard curves were generated using a EV-E LCR4 strain (ATCC VR-248) amplicon, which contains a nucleotide sequence of the polyprotein gene of EV-E, with complementary primers and probes. Amplification of a product was conducted by PCR. The reaction was carried out in a Nexus Gradient thermocycler (Eppendorf, Hamburg, Germany) with a HotStar TaqPlus Master Mix Kit (Qiagen, Hilden, Germany). The 20-μL reaction sample contained 10 μL of HotStar TaqPlus DNA Polymerase (Qiagen), 0.5 μL of each primer at 10 μM (EV-E802 for and EV-E802 rev; Table 1), 7 μL of RNase-free water, and 2 μL of cDNA. The reaction was carried out under the following conditions: the initial denaturation step was at 95 °C for 5 min and was followed by 30 cycles of denaturation at 94 °C for 30 s, primer annealing at 60 °C for 30 s, 72 °C for 60 s and final extension of 72 °C for 10 min. Then, after the PCR products were purified with a Clean-Up Concentrator Kit (A&A Biotechnology, Gdynia, Poland), the amplicon concentration was measured with a BioSpectrometer (Eppendorf, Hamburg, Germany). The gene copy number was calculated based on amplicon concentration and size with a copy number calculator (Genomics and Sequencing Center, University of Rhode Island, Kingston, RI). Standard 10-fold serial dilutions of amplicons (starting at initial dilution to 10^8^ and ending at final dilution to 10^3^) were used as template cDNA.

Intracellular viral RNA detected in enterovirus E-infected untreated (control) cells was assumed to equal 1, and the results obtained from infected lactoferrin-treated cells were expressed as the relative amount of the control virus RNA. Each experiment was repeated three times.

### 4.4. Statistical Analysis

All the results were expressed as the mean values ± standard deviations (SD) of three independent experiments. Data were submitted to one-way analysis of variance (ANOVA). Tukey’s posttest was used to determine differences between control and lactoferrin-treated cells. Statistical evaluation of the results was performed using GraphPad Prism 7 software.

## 5. Conclusions

In summary, the present data indicate that bLF has an antiviral effect on bovine enterovirus but with slightly different mechanisms than those described for human enteroviruses. We did not observe the protective effect of lactoferrin towards cells, although the protein inhibited the replication of the virus at both the adsorption and post-adsorption stages and decreased the viral RNA load in the cells. More detailed studies are needed to determine the mechanism underlying the activity of bovine lactoferrin against enterovirus E. However, there is no doubt that this highly biocompatible protein ensures some degree of protection against infection with bovine enterovirus, which can be of particular importance for young animals that receive this protein in their mother’s milk.

## Figures and Tables

**Table 1:** Primer sequences used for the detection of intracellular enterovirus E RNA.

Primer	Primer Sequence (5′–3′)	Amplicon Size	GenBank Accession No.
EV-E802 for	AAAGGGGGCTGTCGAAACCA	802	DQ092769.1
EV-E 802 rev	GCTAGTGGGCTCAGACTCCG		
EV-E 183 for	TACGCCTTTCGTGGCTTGGA	183	
EV-E 183 rev	TTGCTTTTCCTGGCTTGCCG		

## Data Availability

All data generated and analyzed during this study are available from the corresponding author on reasonable request.

## Sample Availability

Samples of the compounds are available from the authors.
